# The Current Role of Androgen Deprivation in Patients Undergoing Dose-Escalated External Beam Radiation Therapy for Clinically Localized Prostate Cancer

**DOI:** 10.1155/2012/280278

**Published:** 2012-04-24

**Authors:** Michael J. Smith, Naveed H. Akhtar, Scott T. Tagawa

**Affiliations:** ^1^Department of Radiation Oncology, Stitch Radiation Center, Weill Cornell Medical College, 525 East 68th Street, P.O. Box 575, New York, NY 10065, USA; ^2^Division of Hematology & Medical Oncology, Weill Cornell Medical College, 525 East 68th Street, New York, NY 10065, USA; ^3^Department of Urology, Weill Cornell Medical College, 525 East 68th Street, New York, NY 10065, USA

## Abstract

*Purpose*. To review existing literature on the role of androgen deprivation therapy (ADT) with dose escalated radiation therapy. *Methods and Materials*. A PubMed search was undertaken to identify relevant articles. *Results*. Multiple recent studies were identified examining the role of ADT in the current era of radiation dose-escalation. Among the reviewed studies, varying radiation doses and techniques, ADT regimens, and patient selection criteria were utilized. Conflicting results were reported, with some studies demonstrating a benefit of delivering a higher radiation dose with ADT. Other studies failed to show significant benefits with the addition of ADT to dose-escalated RT. *Conclusions*. The benefit of adding ADT to dose-escalated RT is still uncertain. Prospective randomized trials, several of which are ongoing, are necessary to more adequately examine this issue. In the interim, physicians and patients should continue to utilize the existing data to weigh the risks and benefits of each approach to therapy.

## 1. Introduction

Significant improvements in outcome have been achieved in the treatment of prostate cancer (PC) over the last couple of decades. Advancements in external beam technology, such as intensity-modulated radiation therapy (IMRT) and image-guided radiation therapy (IGRT) have allowed for dose escalation, with improvements in biochemical failure and rate of distant metastases (though no overall survival (OS) benefits have yet been demonstrated), without an accompanying increase in short- and intermediate-term toxicity [1–3]. We have also learned through multiple phase III randomized trials that the addition of ADT to external beam radiation therapy (EBRT) in unfavorable or locally-advanced cancers leads to improvements in disease-free survival (DFS), and prostate cancer-specific survival, as well as in OS (Table 1) [4–14]. However, in all of these studies, radiation doses ≤70 Gy were delivered, which are below the current standard doses used. In addition, it is increasingly recognized that ADT may be associated with acute and long-term toxicity. One controversy that currently exists in the radiation community is the role of ADT with dose-escalated radiation therapy in intermediate-risk and high-risk patients. This paper is intended to summarize existing data looking at this issue. In addition, a brief review of toxicity of hormonal therapy will be discussed.

## 2. Materials and Methods

A PubMed literature search was undertaken, and relevant articles were reviewed. Search words and phrases included “dose-escalated radiation therapy and hormones,” “radiation therapy and hormones,” “radiation dose escalation”, “androgen deprivation therapy,” and “hormone toxicity”. English language articles that were relevant to this particular review were summarized in the paper. Two tables were created, summarizing several major past and ongoing randomized studies looking at efficacy and toxicity of radiation therapy and hormonal therapy in the treatment of prostate cancer patients. For the purpose of this discussion, “dose escalation” refers to doses above those used in the landmark ADT and RT trials reviewed in Table 1 (>70 Gy). We focused on the evaluation of dose-escalated EBRT as primary PC therapy, and do not report results of other means of radiation dose-escalation, that is, dose escalation with brachytherapy, a combination of EBRT plus brachytherapy, or adjuvant or salvage RT studies.

## 3. Results

As can be seen in Table 1, multiple phase III randomized trials have demonstrated improvements in DFS, prostate cancer-specific survival, and OS with the addition of ADT to EBRT in locally advanced cancers as well as unfavorable, localized PC. Of note, the RT doses in these trials were all ≤70 Gy. The studies summarized below evaluate the role of ADT when doses >70 Gy were delivered.

The UK Medical Research Council (MRC) reported initial results of their large, multicenter randomized RT01 trial in 2007 [15]. 843 men with cT1-3N0 PC were randomized to 64 Gy or an escalated dose of 74 Gy with neoadjuvant and concurrent androgen suppression (3–6 months, left to physician discretion). In the higher-dose group, there was an advantage in biochemical progression-free survival (bPFS) (71% versus 60%). The prescribed dose was lower than that used in other dose escalation trials [1–3], but it was shown that neoadjuvant androgen deprivation (nADT) did not negate the value of dose escalation. The authors recommend combined modality therapy for the treatment of intermediate and high-risk PC. The duration of hormonal ablation was left to the discretion of the treating physician; therefore, no definitive recommendations can be made regarding length of neoadjuvant ADT. 

Zelefsky et al. [16] analyzed the long-term outcomes of patients treated for clinical T3 disease with three-dimensional conformal radiation therapy (3DCRT). Among patients treated with ≥81 Gy and short-course ADT (6 months), excellent five- and ten-year local control rates of 96% and 88% were attained. In addition, 5 year survival outcomes were at least as favorable as those reported in trials in which longer courses of ADT were used [17]. In RTOG 92–02, long-term ADT resulted in improved outcomes versus short-term treatment, including a survival advantage in patients with high-grade disease. In a Practice Point review of RTOG 92–02, Kollmeier and Zelefsky question whether all patients with locally advanced disease would benefit from long-term ADT in the era of dose escalation. Randomized trials with dose escalation using risk stratification are necessary to help clarify these issues [17].

Three Spanish studies have addressed dose-escalated radiation therapy and ADT [18–20]. Zapatero et al. [18], in a multi-center study, examined risk-adapted ADT with escalated 3DCRT. Seventy-five intermediate-risk patients received neoadjuvant ADT, 4–6 months before and during 3DCRT, and 160 high-risk patients received neoadjuvant and adjuvant ADT (aADT) 2 years after 3DCRT in a nonrandomized fashion. When stratified by treatment group, higher radiation dose (≥72 Gy versus <72 Gy) was significantly associated with an improvement in biochemical DFS (bDFS) in high-risk patients, all of whom received long-term ADT. The 5-year bDFS for high-risk patients treated with neoadjuvant and adjuvant ADT was 63% for RT doses less than 72 Gy and 84% for doses ≥72 Gy (*P* = 0.03). In intermediate-risk patients, an improvement in bDFS was evident with higher RT doses as well (94% versus 56%), but the sample size was small, and the results were not statistically significant (*P* = 0.119) at the timepoint analyzed. In addition, on multivariate analysis, separating intermediate- and high-risk patients, controlling for elective nodal radiation, results confirmed an independent benefit of higher radiation dose for high-risk patients (*P* = 0.021). Despite the inherent limitations of this prospective, non-randomized study, data derived from univariate, and multivariate analysis lend credence to the idea that the use of ADT in high risk patients does not preclude the need for dose escalation. 

In a single-institution review, in high risk patients, a 5-year bDFS of 77% was achieved with a combination of high-dose RT and nADT + aADT; more specifically, 5 year bDFS of >90% was achieved for high risk patients treated with nADT, aADT, an RT dose >72 Gy [19]. In a more recent report, in a retrospective analysis of 137 high risk patients treated with long-term ADT + RT to a dose of >78 Gy, with median followup of 48 months, an actuarial 5 year bDFS of 97.8% was reported [20]. These results, although preliminary, compare favorably with results from series in which ADT is combined with conventional dose RT. Again, these results seem to add weight to the notion that the use of ADT in high risk patients does not preclude the need for dose escalation in these patients. As prospective studies are needed to confirm these findings, the cooperative Spanish group of Clinical Research in Radiation Oncology has activated a Phase III, multi-center, randomized trial looking at the role and duration of ADT when combined with high-dose RT in intermediate and high risk patients [21].

Given improvements in outcome seen in dose escalation studies and the less certain need for long-term hormonal therapy, investigators from the University of Chicago retrospectively reviewed outcomes of intermediate and high-risk patients treated with RT and short-term ADT [22]. Median RT dose was 74 Gy; 60% received a dose ≥74 Gy and 55% were treated with IMRT. All patients received ADT for 1–6 months (median, 4 months), typically starting 2 months before RT. Given the small number of patients with biochemical failure in the intermediate risk category, it was felt difficult to make conclusions or recommendations, such as identifying a subset of patients for which ADT is unnecessary. In the high risk category, patients with locally confined disease treated with dose-escalated RT had the best outcomes (4-year freedom from biochemical failure of 77% in pts with T1-T2c disease receiving greater than or equal to 74 Gy). However, patients with clinical T3 disease had less favorable outcomes. Therefore, short-term ADT with RT may not be sufficient therapy in this group. As the contemporary high-risk patient often has locally-confined disease and will be treated with dose-escalated RT, the authors feel that long-term ADT may not be warranted in all high risk patients and suggest that selected high risk patients may be reasonably treated with short-term ADT + doses ≥74 Gy to help reduce the potential side effects of long-term ADT. Limitations of the study include the retrospective nature of the study and the heterogeneous patient characteristics. Additional prospective studies are necessary to confirm these findings. 

Valicente et al. evaluated the effect of adding ADT to dose escalated RT, analyzing data from the RTOG 94–06 Phase I/II dose-escalation trial [23]. ADT was combined with RT doses exceeding 73.8 Gy (mean 78.5 Gy, maximum 84.3 Gy). To limit the rectal volume in the high-dose region, the minimum planning target volume (PTV) dose was limited to 73.8 Gy, whereas the minimum gross tumor volume dose was prescribed to 79.2 Gy in 44 fractions. For this analysis, only patients receiving a minimum PTV dose of greater than 73.8 Gy were considered. ADT was generally started 2-3 months before RT, and continued for a longer duration in high risk patients. When patients were stratified by risk groups, no significant effect on bNED or DFS was noted with the addition of ADT to HDRT. When adjusting for higher PSA (greater than 20) and for higher Gleason scores (>7), there was no obvious benefit for high risk patients when long-term ADT was added to EBRT at doses greater than 73.8 Gy, although there was a strong trend (*P* = 0.0507). The nonrandomized nature of the study and the use of variable courses and durations of ADT limit conclusions, though the study adds to the pool of data justifying prospective, randomized trials examining this issue.

In a recent publication from the William Beaumont Hospital group, Krauss et al. looked at the potential benefits of adding ADT to high dose radiation therapy (HDRT) [24]. The title of the paper aptly summarizes their conclusions: “Lack of Benefit for the Addition of Androgen Deprivation Therapy to Dose-Escalated Radiotherapy in the Treatment of Intermediate and High-Risk Prostate Cancer.” 1044 intermediate (*n* = 782) and high risk (*n* = 262) patients were treated with HDRT, in the form of EBRT alone, brachytherapy (high or low dose rate (HDR, LDR, respectively)), or HDR plus pelvic EBRT. Looking at the group of patients treated with EBRT only, dose was prescribed as a minimum to the PTV, at a median level of 75.6 Gy (median isocenter dose 79.6 Gy). ADT was given at the discretion of the treating physician. This analysis looked at outcomes for those patients receiving ADT versus those who did not. Of note, patients receiving ADT had higher median PSA and mean biopsy scores as well as a greater number of positive biopsy cores. The median and mean hormonal therapy duration was 6 months and 9.8 months, respectively.

Four hundred sixty-nine patients (365 intermediate, 104 high risk) were treated with EBRT without brachytherapy with median followup of 4.1 years. No significant difference in 5-year biochemical control was noted in those who did or did not receive ADT (81.2% and 84.8%, respectively). Five-year freedom from clinical failure rates of 96.4% and 98.6% and 5-year freedom from distant metastases (97.7% with versus 99.2% without ADT; *P* = 0.03) favored the patients who did not receive ADT. No difference in OS was appreciated. No significant differences in any of the clinical endpoints persisted when analyzed separately for the intermediate and high-risk groups, though there was a trend towards a survival advantage for those with clinical stage ≥T2a treated with ADT + HDRT, despite a small number of events (*n* = 5). Limitations of this study include selection bias, with the choice to administer ADT left to the discretion of the investigator (and patient). 

In another recent report, Tendulkar et al. [25] evaluated 585 high risk patients treated with image-guided EBRT to doses >74 Gy at the Cleveland Clinic; 95% of these patients received ADT. The mean EBRT dose was 78 Gy (IMRT was used in 73% of patients), and the median ADT duration was 6 months; patients with multiple intermediate risk factors were also considered HR in this report. No correlation was found with the duration of ADT and either bRFS, DMFS, or PCSM in this setting of dose-escalated RT. This study is one of the largest reported analyses evaluating HR patients treated with a combination of modern, dose-escalated EBRT and ADT. The authors comment that the majority of patients received 6 months of ADT, so it is possible that there were too few patients received long-term ADT to detect an effect on outcome. In addition, patients with more unfavorable features received a significantly longer mean duration of ADT. Therefore, given these factors, and the retrospective nature of the study, it is not possible to draw definitive conclusions regarding the optimal duration of ADT, when used with high-dose RT, and prospective studies are needed to clarify this issue. These investigators also suggests that, within the HR group, there may be a more favorable subgroup of HR patients that have an acceptable outcome with short-duration ADT and dose escalated RT. However, they identified an unfavorable subgroup with clinically organ-confined, Gleason score 9 to 10 prostate cancer, or Gleason 8 prostate cancer with a PSA greater than 10 ng/mL, that had a dismal prognosis with dose-escalated EBRT and ADT; they suggest that additional clinical trials investigating more aggressive treatment regimens are necessary.

Stenmark et al. [26] recently reported on 718 patients treated at the University of Michigan Medical Center, analyzing prognostic factors for patients treated with high-dose EBRT (doses of at least 75 Gy) with or without ADT. ADT resulted in a significant improvement in metastases-free survival, clinical progression, and PCSM across multiple definitions of HR disease, even with dose-escalated RT. The number of National Comprehensive Cancer Network (NCCN) HR risk factors predicted for a worse outcome, even in the setting of dose-escalation. It was suggested by the authors that patients with multiple high-risk features or the presence of Gleason 5 pattern be considered for clinical trials investigating novel local and/or systemic therapies. 

Given the lack of prospective, randomized trials evaluating the role of ADT with dose escalation, individualization of therapy is essential. One important factor to consider is the patient's level of comorbidity. In a postrandomization analysis of the Dana-Farber Cancer Institute randomized trial 95-096, in which patients with IR and HR features were treated with 70 Gy (non-dose-escalated RT) with or without 6 months of androgen suppression therapy (AST), an improvement in survival was noted in men with localized prostate cancer with IR or HR disease with the addition of AST. However, in men with moderate-to-severe comorbidity, no benefit was observed in either patients with IR or HR disease [27]. The authors comment that they would be cautious about using AST in men with moderate to severe comorbidity, as they may not benefit, and, in this study, even had worse survival with the addition of AST (although this was not statistically significant). The concern is that excess cardiovascular deaths with the addition of AST may negate any PCSS benefit from AST. While this issue remains controversial, a recent meta-analysis of randomized trials [28] evaluating nonmetastatic, unfavorable-risk prostate cancer, did not reveal an increase in cardiovascular death with ADT, but did reveal a lower risk of PCSM and all-cause mortality, supporting combination therapy as standard of care evidence-based practice.

## 4. Discussion

In the contemporary era of dose-escalated radiation therapy, the clear role of androgen deprivation remains undefined. In this review of data from recent, mostly nonrandomized and/or retrospective studies examining this issue, using nonstandardized criteria, such as varying radiation doses, and techniques, and varying ADT regimens (often left to individual physician discretion), conflicting results have been reported. In the UK MRC RT01 trial [15], and evaluating the Spanish data [18–20], the use of ADT did not preclude the use of higher-RT doses, demonstrating a benefit of delivering a higher RT dose with ADT. Stenmark et al. [26], again reviewed above, reported that ADT resulted in a significant improvement in metastases-free survival, and PCSM across multiple definitions of HR disease, even with dose-escalated RT. In two of the other reviewed articles [23, 24], conclusions drawn from the available data suggest that addition of ADT to dose-escalated RT may not be necessary. Again, these were not-randomized analyses, with their inherent biases, using non-standardized ADT courses and without long-term followup. However, they are all large studies, evaluating large numbers of patients. Given the lack of available randomized, prospective data, these results do need to be considered in individualizing treatment for our patients. 

In addition to the studies reviewed above, Zelefsky et al. reported excellent results in the treatment of T3 disease with shorter-term ADT with high-dose RT (≥81 Gy) [29]. While comparing nonrandomized results is fraught with problems, it may be that the use of higher-dose RT at MSKCC could have contributed to the better outcomes than those reported by the Chicago group (using lower doses of RT) for cT3 pts, in the setting of short-term ADT. In the recent report from Tendulkar et al. [25], reviewed above, the duration of ADT, when added to modern, dose escalated EBRT in HR patients, did not correlate with outcomes (again, noting the limitations of this retrospective study, mentioned above). Prospective, randomized trials evaluating length of ADT with standardized dose-escalated RT doses are necessary to provide more definitive answers. 

In addition to the use of lower doses (≤70 Gy) in the older trials evaluating ADT plus RT, other significant advances in the current planning and delivery of RT need to be taken into account when assessing the relevancy of the data from the landmark studies presented in Table 1. For instance, many of these studies were designed and conducted in the 1980's and 1990's, when CT treatment planning was not used [23]. Irrespective of dose-escalation, with other technological improvements in radiation therapy, including improved targeting, are the reported benefits from these studies as significant in the contemporary era? It is difficult to compare results from studies from different eras, given changes in the diagnosis (PSA versus pre-PSA era) of PC, differing definitions of biochemical failure, and changes in epidemiology (stage and grade migration) as well as differences in the treatment of prostate cancer over the last few decades [30]. The specific nature of the interaction between ADT and EBRT has not been answered. Some have suggested that the addition of ADT compensated for the inadequate RT doses used in the aforementioned landmark trials (≤70 Gy) [31]. Zelefsky et al. [16] suggest that a relatively short course of ADT combined with high-dose RT “may have a profound effect on tumor control within the prostate,” while longer courses of ADT may be necessary to further decrease the risk of distant metastases.

Hormonal therapy for PC is one of the most effective types of systemic therapies in solid tumor malignancies and represents one of the first examples of “targeted therapy” [32]. However, we have become increasingly aware of the adverse events associated with ADT. Some of these toxicities can have deleterious effects on quality of life, and others may contribute to increased risks for serious health concerns. Vasomotor toxicity (hot flashes) and sexual dysfunction are amongst the most well-recognized immediate adverse effects. Additional toxicities related to the metabolic effects of hormonal therapy include bone and muscle changes, lipid and glucose metabolic changes, and cognitive changes are important to recognize. Though most prospective randomized radiation-based studies with or without ADT have not led to an increased number of cardiovascular events, physicians should be aware of far-reaching consequences of androgen deprivation therapy and should incorporate strategies for preventing and managing toxicities into routine practice. 

ADT for PC is a powerful tool in the armamentarium of the uro-oncologist. However, because of increasingly recognized toxicities, indiscriminate use is not warranted. Every physician should maintain a careful balance between risk of potentially dangerous toxicities and beneficial antitumor efficacy for each individual in conjunction with personal preferences.

The optimal treatment for men labeled as intermediate risk remains controversial. This is at least partly due to the heterogeneous patient population represented in this group using the traditional clinical factors of Gleason sum, clinical T stage, and baseline PSA level. Stratifying patients according to whether they have one or more intermediate risk features or using improved prognostic tools such as nomograms in future studies will likely help to clarify the situation. Possibilities for treatment of intermediate risk patients include dose-escalated RT alone (for instance, in a patient who would prefer to avoid the potential side effects of ADT or those with comorbidities decreasing the therapeutic utility of ADT) versus combination short-term (4–6 months) ADT + RT, again individualizing treatment based on number of risk factors, and so forth.

Many groups recommend that standard practice remain a combination of ADT and RT for high-risk disease, given that multiple randomized prospective trials have demonstrated a survival advantage with the addition of ADT to RT (Table 1). The treatment of intermediate-risk disease is more controversial, as only one prospective, randomized trial demonstrated an advantage to adding ADT to RT in this group [7], (of note, high-risk patients were also included and benefited), leading to current recommendations for dose-escalated RT with or without ADT in intermediate risk patients.

There are still no mature prospective, randomized data reported evaluating the role of ADT with dose-escalated RT. However, there are several studies underway (Table 2) [33–36], including 2 RTOG studies that have been designed to address this issue. RTOG 0815, “A Phase III Randomized Trial of Dose-Escalated Radiotherapy with or without Short-Term Androgen Derivation Therapy for Patients with Intermediate-Risk Prostate Cancer,” randomizes pts to dose escalated RT alone versus dose-escalated RT combined with short-term (6 months) androgen blockade (LHRH agonist + antiandrogen). Radiation dose-escalation can be achieved with either dose-escalated EBRT, EBRT + LDR brachytherapy boost, or EBRT + HDR brachytherapy boost in this study. In the dose-escalated EBRT group, the prescribed dose is 79.2 Gy. Patients are stratified by the number of risk factors. RTOG 0924, “Androgen Deprivation Therapy and High Dose Radiotherapy With or Without Whole-Pelvic Radiotherapy in Unfavorable Intermediate or Favorable High Risk Prostate Cancer” [33] is another ongoing randomized phase III trial. This study randomizes men to neoadjuvant ADT + prostate and seminal vesicle RT + boost to prostate and proximal seminal vesicles versus neoadjuvant ADT + whole pelvic RT + boost to prostate and proximal seminal vesicles. The RT boost can be delivered with IMRT or brachytherapy. ADT is given for 6 months (short term) versus 32 months (long term).

## 5. Conclusions

Improvements in technology have led to the ability to safely escalate doses of RT to the prostate, and this has translated into improvements in disease control. Seminal, but historical studies utilizing lower doses of RT demonstrated survival benefits with the addition of ADT to EBRT. The short- and long-term consequences of ADT are being increasingly realized and the benefits of adding ADT to dose-escalated RT remain poorly defined. We need to continue to enroll patients in the available prospective, randomized clinical trials examining these important issues. For those without access or ineligible for studies, ADT + EBRT remains the standard for those with high-risk disease. However for those without access to trials and for intermediate risk (and selected cases of high risk) disease, individualization of therapy, taking into account known prognostic variables, patient comorbidities and preferences, and risks and benefits of varying amounts of ADT, is suggested. This may be most effective in a multidisciplinary fashion, with input from urologists, radiation oncologists, medical oncologists, and primary care physicians, extrapolating from existing data.

## Figures and Tables

**Table 1:** 

Data Source	Type	Arms	Population	Toxicity	Results
RTOG 85–31 [4]	Prospective randomized trial	WPRT + boost to 65–70 Gy (cT3, pT3, LN+)	Total: 189 RT (65–70 Gy) + ADT (indefinite) RT (65–70 Gy) alone	CR of CV death: 11% 14%	10-year OS benefit for all patients; OS benefit for Gleason 7–10 in subset analysis.

RTOG 86–10 [5]	Prospective randomized trial	WPRT + Boost (66–70 Gy) ±2 mos. (nADT) and 2 mos. (cADT). (Locally advanced disease).	Total: 471 RT + ADT (4 mos) RT (alone).	CR of CV death: 14% 11%	Significant benefit in ↓DM, ↑CSS, and ↑DFS, OS advantage for Gleason score <7.

RTOG 92–02 [6]	Prospective randomized trial	WPRT + Boost to 65–70 Gy + 2 mos. nADT + 2 mos. cADT ± 2 year aADT	Total: 1554 RT + ADT (28 mos.) RT + ADT (4 mos.)	CR of CV death: 13.5% 11%	Survival advantage for pts. with Gleason score 8–10.

D'Amico et al. [7, 8]	Prospective randomized trial	45 Gy (prostate and seminal vesicles) + boost to 70 Gy ± 6 mos. ADT (nADT, cADT, or aADT).	Total: 206 RT + nADT or cADT or ADT RT (alone)	Age: >65 yrs. 6 mos. HT Fatal MIs. 7% 5-6%	OS advantage for pts. with hormonal manipulation with minimal or no comorbidities.

RTOG 94–13 [9]	Prospective randomized trial	70.2 Gy (50.4 to WP if on WP arms). 4 arms: WPRT + nADT PORT + nADT WPRT + aADT PORT + aADT	Total: 1279 WPRT + nADT + Boost (*n* = 320) PORT + nADT (*n* = 319) WPRT + aADT (*n* = 319) PORT + aADT (*n* = 321)	Acute radiation toxicity: WPRT + nADT = (8%) PORT + nADT = (5%) WPRT + ADT = (3%) PORT + ADT = (3%). Grade 3 GI toxicity: WPRT + nADT = 5% PORT + nADT = 1% WPRT + ADT = 2% PORT + ADT = 2%	Improved PFS in WPRT + nADT arm as compared to others.

TROG 9601 [10]	Prospective randomized trial	66 Gy + 0 versus 3 versus 6 mos. nADT (T2b-T4).	Total: 818 3 arms: RT (alone) RT + 3 mos. nADT RT+ 6 mos. nADT		Improvement in 5-year LF, bFFS, and DFS, freedom from salvage with 3 or 6 mos nADT.

EORTC 22961 [11]	Prospective randomized trial	70 Gy (50 Gy WPRT) + 6 mos. cADT versus 3 yrs. on aADT	Total: 970 WPRT + 6 mos. cADT WPRT + 3 yrs. aADT	No difference in fatal cardiac events (3-4%). More hot flushes and ↓ sexual function	3-year ADT improved overall mortality 19 versus 15.2%.Prostate cancer mortality: 4.7% versus 3.2%.

RTOG 94–08 [12]	Prospective randomized trial	66 Gy ± 2 mos. nADT + 2 mos. cADT	Total: 1989 RT (alone) RT + nADT (2 mos.) + cADT (2 mos.)	Risk of acute, late GU, GI, and hemat. Toxicities is same in both arms. Grade 4 < 3% Grade 5 < 1%	Short-term ADT before and during RT was associated with significantly decreased DSM and increased OS for IR pts.

EORTC 22863 [13]	Prospective randomized trial	70 Gy (50 Gy WPRT) ± 3 year goserelin starting on first day of RT. (T3-T4 or T1-T2 Gleason > 7)	Total: 415 RT + Goserelin (aADT) RT (alone)	No difference in 10 year cardiac mortality (8–11%)	OS and DFS benefit for patients on combined therapy arm.

Crook et al. [14]	Prospective randomized trial	3 or 8 mos. of flutamide or goserelin before 66 Gy RT	Total: 378 Flutamide or goserelin (3 mos.) + RT Flutamide or goserelin (8 mos.) + RT	—	5-year DFS improvement for high-risk patients in the 8 mos. arm.

Nguyen et al. [28]	Meta-analysis of 8 prospective randomized trials	Nonmetastatic unfavourable risk PC pts ± ADT	Total: 4141 Nonmetastatic PC + ADT Control group	ADT use is not associated with an increased risk of CVD	ADT is associated with a lower risk of PCSM and all-cause mortality.

RTOG: Radiation therapy oncology group, WPRT: whole pelvic radiation therapy, RT: Radiation therapy, mos.: months, ADT: Androgen deprivation therapy, CR: Cumulative risk, CV: Cardiovascular, OS: Overall survival, nADT: Neo-adjuvant androgen deprivation therapy, cADT: Concomitant androgen deprivation therapy, aADT: Adjuvant androgen deprivation therapy, DM: Distant metastases, DSM-Disease specific mortality, DFS: Disease free survival, MI: Myocardial infarction, PORT: Prostatic bed only radiation therapy, LF: local failure, bFFS: Biochemical failure free survival, EORTC: European organization for research and treatment of cancer, TROG: Trans-Tasman Radiation Oncology Group, CVD: Cardiovascular disease related death.

**Table 2:** Current Trials.

Trial	Arms	Population	Objective
Zapatero et al. [21]	76 Gy (HDRT) + 4 mos. (nADT+cADT) or HDRT + 2 yrs. aADT	Planned enrollment 358 (preliminary results reported in 298 pts [21]; Intermediate and high-risk PC	Primary endpoints are biochemical disease-free survival and toxicity scores.

RTOG 08–15 [37]	Dose-escalated RT ± ADT (intermediate risk disease)	Planned enrollment 1520; Intermediate risk PC	Primary end-point is OS with secondary end point of acute and late toxicities.

RTOG 09–24 [33]	ADT + RT (high dose) ± WPRT in unfavorable intermediate or favorable high-risk pts.	Planned enrollment 2580; Intermediate and high risk PC	OS of patients treated with ADT and RT versus ADT and WPRT.

NCT00967863 [34]	80 Gy + ADT vs 70 Gy RT + ADT	Planned enrollment 500; High risk PC	The trial is primarily designed to look at biochemical or clinical progression-free survival at 5 years. OS, CSS, and toxicity are the secondary end points of the study.

NCT00223145 [35]	RT (dose escalation from 70–76 Gy) + nADT and cADT or RT (76 Gy) + nADT and cADT in IR PC pts.	Planned enrollment 600; Intermediate risk PC	This is an efficacy study looking at biochemical failure and DFS. The secondary endpoints are OS and toxicity.

NCT00104741 [36]	80 Gy conformal RT ± nADT and cADT	Planned enrollment 450; Intermediate risk PC	Primary end-point: efficacy, 5-year-survival, OS and toxicity.

HDRT: High dose radiation therapy, mos.: Months, yrs.: Years, ADT: Androgen deprivation therapy, nADT: Neo-adjuvant androgen deprivation therapy, cADT: Concomitant androgen deprivation therapy, aADT: Adjuvant androgen deprivation therapy, OS: Overall survival, RTOG: Radiation therapy oncology group, PBRT: Radiation therapy to prostate bed only, wks.: weeks,STADT: Short term androgen deprivation therapy, LTADT: Long term androgen deprivation therapy PLNRT: Pelvic lymph node radiation therapy, LN: Lymph node, WPRT: Whole pelvic radiation therapy, 3D-CRT: 3-Dimensional conformal radiation therapy, IMRT: Intensity modulated radiation therapy, DFS: Disease free survival.

## References

[1] Kuban DA, Tucker SL, Dong L (2008). M.D. Anderson randomized dose-escalation trial for prostate cancer. *International Journal of Radiation Oncology Biology Physics*.

[2] Zietman AL, DeSilvio ML, Slater JD (2005). Comparison of conventional-dose vs high-dose conformal radiation therapy in clinically localized adenocarcinoma of the prostate: a randomized controlled trial. *Journal of the American Medical Association*.

[3] Peeters STH, Heemsbergen WD, Koper PCM (2006). Dose-response in radiotherapy for localized prostate cancer: results of the Dutch multicenter randomized phase III trial comparing 68 Gy of radiotherapy with 78 Gy. *Journal of Clinical Oncology*.

[4] Efstathiou JA, Bae K, Shipley WU (2009). Cardiovascular mortality after androgen deprivation therapy for locally advanced prostate cancer: RTOG 85-31. *Journal of Clinical Oncology*.

[5] Pilepich MV, Winter K, John MJ (2001). Phase III radiation therapy oncology group (RTOG) trial 86-10 of androgen deprivation adjuvant to definitive radiotherapy in locally advanced carcinoma of the prostate. *International Journal of Radiation Oncology Biology Physics*.

[6] Hanks GE, Pajak TF, Porter A (2003). Phase III trial of long-term adjuvant androgen deprivation after neoadjuvant hormonal cytoreduction and radiotherapy in locally advanced carcinoma of the prostate: the Radiation Therapy Oncology group Protocol 92-02. *Journal of Clinical Oncology*.

[7] D’Amico AV, Manola J, Loffredo M, Renshaw AA, DellaCroce A, Kantoff PW (2004). 6-Month androgen suppression plus radiation therapy vs radiation therapy alone for patients with clinically localized prostate cancer: a randomized controlled trial. *Journal of the American Medical Association*.

[8] D’Amico AV, Chen MH, Renshaw AA, Loffredo M, Kantoff PW (2008). Androgen suppression and radiation vs radiation alone for prostate cancer: a randomized trial. *Journal of the American Medical Association*.

[9] Lawton CA, DeSilvio M, Roach M (2007). An update of the phase III trial comparing whole pelvic to prostate only radiotherapy and neoadjuvant to adjuvant total androgen suppression: updated analysis of RTOG 94-13, with emphasis on unexpected hormone/radiation interactions. *International Journal of Radiation Oncology Biology Physics*.

[10] Denham JW, Steigler A, Lamb DS (2005). Short-term androgen deprivation and radiotherapy for locally advanced prostate cancer: results from the Trans-Tasman Radiation Oncology Group 96.01 randomised controlled trial. *Lancet Oncology*.

[11] Bolla M, De Reijke TM, Van Tienhoven G (2009). Duration of androgen suppression in the treatment of prostate cancer. *New England Journal of Medicine*.

[12] Jones CU, Hunt D, McGowan DG (2011). Radiotherapy and short-term androgen deprivation for localized prostate cancer. *New England Journal of Medicine*.

[13] Bolla M, Van Tienhoven G, Warde P (2010). External irradiation with or without long-term androgen suppression for prostate cancer with high metastatic risk: 10-year results of an EORTC randomised study. *The Lancet Oncology*.

[14] Crook J, Ludgate C, Malone S (2009). Final report of multicenter Canadian Phase III randomized trial of 3 versus 8 months of neoadjuvant androgen deprivation therapy before conventional-dose radiotherapy for clinically localized prostate cancer. *International Journal of Radiation Oncology Biology Physics*.

[15] Dearnaley DP, Sydes MR, Graham JD (2007). Escalated-dose versus standard-dose conformal radiotherapy in prostate cancer: first results from the MRC RT01 randomised controlled trial. *Lancet Oncology*.

[16] Zelefsky MJ, Yamada Y, Kollmeier MA, Shippy AM, Nedelka MA (2008). Long-term outcome following three-dimensional conformal/intensity-modulated external-beam radiotherapy for clinical stage T3 prostate cancer. *European Urology*.

[17] Kollmeier MA, Zelefsky MJ (2008). What is the role of androgen deprivation therapy in the treatment of locally advanced prostate cancer?. *Nature Clinical Practice Urology*.

[18] Zapatero A, Valcárcel F, Calvo FA (2005). Risk-adapted androgen deprivation and escalated three-dimensional conformal radiotherapy for prostate cancer: does radiation dose influence outcome of patients treated with adjuvant androgen deprivation? A GICOR study. *Journal of Clinical Oncology*.

[19] Zapatero A, Ríos P, Marín A, Mínguez R, García-Vicente F (2006). Dose escalation with three-dimensional conformal radiotherapy for prostate cancer. Is more dose really better in high-risk patients treated with androgen deprivation?. *Clinical Oncology*.

[20] Zapatero A, García-Vicente F, Martín De Vidales C (2011). Long-term results after high-dose radiotherapy and adjuvant hormones in prostate cancer: how curable is high-risk disease?. *International Journal of Radiation Oncology Biology Physics*.

[21] Zapatero A, Guerrero A, Maldonado X (2010). Long-term versus short-term androgen deprivation combined with high-dose radiotherapy for localized prostate cancer: a Spanish multicenter phase III trial. *Journal of Clinical Oncology *.

[22] Liauw SL, Stadler WM, Correa D, Weichselbaum RR, Jani AB (2010). Dose-escalated radiotherapy for high-risk prostate cancer: outcomes in modern era with short-term androgen deprivation therapy. *International Journal of Radiation Oncology Biology Physics*.

[23] Valicenti RK, Bae K, Michalski J (2011). Does hormone therapy reduce disease recurrence in prostate cancer patients receiving dose-escalated radiation therapy? An analysis of Radiation Therapy Oncology Group 94-06. *International Journal of Radiation Oncology Biology Physics*.

[24] Krauss D, Kestin L, Ye H (2010). Lack of benefit for the addition of androgen deprivation therapy to dose-escalated radiotherapy in the treatment of intermediate- and high-risk prostate cancer. *International Journal of Radiation Oncology, Biology, Physics*.

[25] Tendulkar RD, Reddy CA, Stephans KL (2012). Redefining high-risk prostate cancer based on distant metastases and mortality after high-dose radiotherapy with androgen deprivation therapy. *Int J Radiat Oncol Biol Phys*.

[26] Stenmark MH, Blas K, Halverson S, Sandler HM, Feng FY, Hamstra DA (2011). Continued benefit to androgen deprivation therapy for prostate cancer patients treated with dose-escalated radiation therapy across multiple definitions of high-risk disease. *International Journal of Radiation Oncology Biology Physics*.

[27] Nguyen PL, Chen MH, Beard CJ (2010). Radiation with or without 6 months of androgen suppression therapy in intermediate and high risk clinically localized prostate cancer: a postrandomization analysis by risk group. *International Journal of Radiation Oncology Biology Physics*.

[28] Nguyen PL, Je Y, Schutz FAB (2011). Association of androgen deprivation therapy with cardiovascular death in patients with prostate cancer: a meta-analysis of randomized trials. *Journal of the American Medical Association*.

[29] Zelefsky MJ, Chan H, Hunt M, Yamada Y, Shippy AM, Amols H (2006). Long-Term Outcome of High Dose Intensity Modulated Radiation Therapy for Patients With Clinically Localized Prostate Cancer. *Journal of Urology*.

[30] Nanda A, D’Amico AV (2008). Combined radiation and hormonal therapy or dose escalation for men with unfavourable-risk prostate cancer: an evidence-based approach using a synthesis of randomized clinical trials. *British Journal of Urology International*.

[31] Speight JL, Roach III M (2005). Radiotherapy in the management of clinically localized prostate cancer: evolving standards, consensus, controversies and new directions. *Journal of Clinical Oncology*.

[32] Huggins C, Hodges CV (1941). Studies on prostatic cancer. I. The effect of castration, of estrogen and of androgen injection on serum phosphatases in metastatic carcinoma of the prostate. *Cancer Research*.

[33] http://clinicaltrials.gov/ct2/show/NCT01368588/.

[34] http://clinicaltrials.gov/ct2/show/NCT00967863/.

[35] http://clinicaltrials.gov/ct2/show/NCT00223145/.

[36] http://www.clinicaltrials.gov/ct2/show/NCT00104741/.

[37] http://clinicaltrials.gov/ct2/show/NCT00936390/.

